# *Babesia microti*: Pathogen Genomics, Genetic Variability, Immunodominant Antigens, and Pathogenesis

**DOI:** 10.3389/fmicb.2021.697669

**Published:** 2021-09-03

**Authors:** Ankit Puri, Surabhi Bajpai, Scott Meredith, L. Aravind, Peter J. Krause, Sanjai Kumar

**Affiliations:** ^1^Laboratory of Emerging Pathogens, Division of Emerging and Transfusion Transmitted Diseases, Office of Blood Research and Review, Center for Biologics Evaluation and Research, Food and Drug Administration, Silver Spring, MD, United States; ^2^Department of Bioscience and Biotechnology, Banasthali Vidyapith, Banasthali, India; ^3^National Center for Biotechnology Information, National Library of Medicine, National Institutes of Health, Bethesda, MD, United States; ^4^Department of Epidemiology of Microbial Diseases, Yale School of Public Health and Yale School of Medicine, New Haven, CT, United States

**Keywords:** *Babesia microti*, biomarkers, genomics, pathogen, population genetics, tick-borne diseases

## Abstract

More than 100 *Babesia* spp. tick-borne parasites are known to infect mammalian and avian hosts. *Babesia* belong to Order Piroplasmid ranked in the Phylum Apicomplexa. Recent phylogenetic studies have revealed that of the three genera that constitute Piroplasmida, *Babesia* and *Theileria* are polyphyletic while *Cytauxzoon* is nested within a clade of *Theileria*. Several *Babesia* spp. and sub-types have been found to cause human disease. *Babesia microti*, the most common species that infects humans, is endemic in the Northeastern and upper Midwestern United States and is sporadically reported elsewhere in the world. Most infections are transmitted by Ixodid (hard-bodied) ticks, although they occasionally can be spread through blood transfusion and rarely via perinatal transmission and organ transplantation. Babesiosis most often presents as a mild to moderate disease, however infection severity ranges from asymptomatic to lethal. Diagnosis is usually confirmed by blood smear or polymerase chain reaction (PCR). Treatment consists of atovaquone and azithromycin or clindamycin and quinine and usually is effective but may be problematic in immunocompromised hosts. There is no human *Babesia* vaccine. *B. microti* genomics studies have only recently been initiated, however they already have yielded important new insights regarding the pathogen, population structure, and pathogenesis. Continued genomic research holds great promise for improving the diagnosis, management, and prevention of human babesiosis, and in particular, the identification of lineage-specific families of cell-surface proteins with potential roles in cytoadherence, immune evasion and pathogenesis.

## Introduction

**FIGURE 1:**
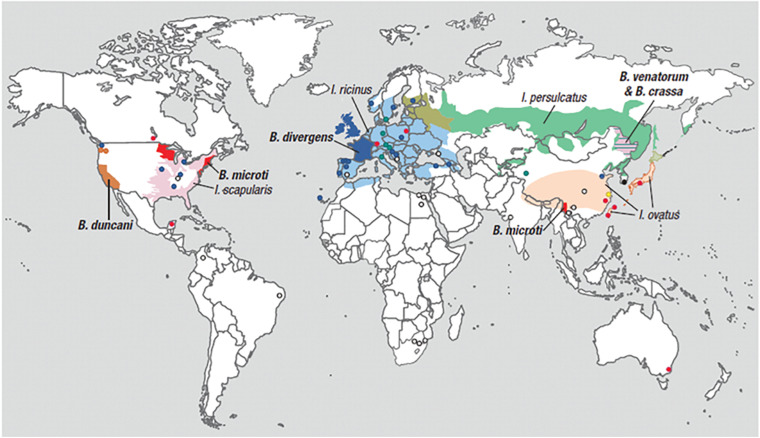
Geographic range of *Ixodes*-tick vectors and location of human babesiosis reports. The *lighter colors* represent the geographical range of several *Ixodes* tick species known or suspected to transmit *Babesia* parasites to humans but where human babesiosis is rare (<5 cases), unreported or absent. *Ixodes ricinus* and *Ixodes persulcatus* are sympatric in southern Finland, Estonia, Latvia, and northwestern Russia, while *I. persulcatus* and *Ixodes ovatus* are both enzootic in Hokkaido in northern Japan. The *darker colors* represent regions where human babesiosis is endemic or sporadic (defined by ≥5 cases). *Circles* indicate single cases, except in three locations (Mexico, Montenegro, and eastern Poland) where all patients at these locations were reported from the same hospital. *Colors* indicate *Babesia* species: red for *B. microti*, orange for *B. duncani*, blue for *B. divergens*, green for *B. venatorum*, pink for *B. crassa* like agent, black for KO-1, yellow for *Babesia* spp. XXB/HangZhou, and white for undetermined *Babesia* spp. The map does not show asymptomatic cases and travel-associated cases [adapted from Vannier and Krause (2012) and (Ryan et al., 2019) from The New England Journal of Medicine, Edouard Vannier, PhD., and Peter J. Krause, M. D., Human Babesiosis, 366, 2399. Copyright (2012) Massachusetts Medical Society. Reprinted with permission.

Human babesiosis is an emerging tick-borne disease caused by several *Babesia* species found in the temperate regions of the world. *Babesia* and related *Theileria* organisms are intraerythrocytic parasites that belong to the same phylum (Apicomplexa) as *Plasmodia* with which they share several microbiologic and pathogenic features. Among the many *Babesia* species that can infect humans, *Babesia microti* is the most prevalent and the primary cause of human babesiosis. Disease due to this pathogen is endemic in the Northeastern and northern Midwestern United States and reported sporadically in other temperate regions of the world (Figure 1) (Homer et al., 2000; Matsui et al., 2000; Hunfeld et al., 2008; Vannier and Krause, 2012; Zhou et al., 2014; Liu et al., 2017). *B. microti* is primarily transmitted by *Ixodes scapularis* ticks but also through blood transfusion, organ transplantation, and perinatally (Fox et al., 2006; Herwaldt et al., 2011; Brennan et al., 2016). Other *Babesia* spp. that are known to infect humans include *Babesia crassa-*like agent, *Babesia divergens*, *B. divergens*-like agent, *Babesia duncani*, *B. microti*-like agent, *Babesia motasi*, and *Babesia venatorum*. A comprehensive list of *Babesia* spp. that are reported to cause human babesiosis, their areas of transmission, reservoir hosts and tick-vectors are provided in Table 1.

**TABLE 1:** *Babesia* that cause human babesiosis.

*Babesia* species	Year Discovered/Reported	Major Region of Transmission	Primary vector
*Babesia microti*	1968^a^	United States (Northeast, northern Midwest)	*Ixodes scapularis*
*Babesia divergens*	1957^b^	Western Europe	*Ixodes ricinus*
*Babesia duncani*	1991^c^	United States (Farwest)	*Dermacentor albipictus*
*Babesia venatorum*	2003^d^	Europe, China	*I. ricinus* and *Ixodes persulcatus*
*Babesia motasi*	2007^e^	South Korea	Unknown
*Babesia crassa*-like agent	2018^f^	Northeast China	*I. persulcatus*
*Babesia divergens*-like	1996^g^	United States	Unknown
*Babesia microti*-like	1997^h^	Taiwan, Japan	Unknown

*^*a*^Scholtens et al. (1968); ^*b*^Skrabalo and Deanovic (1957) (probable *B. divergens*); ^*c*^Quick et al. (1993); ^*d*^Herwaldt et al. (2003); ^*e*^Kim et al. (2007); ^*f*^Jia et al. (2018); ^*g*^Herwaldt et al. (1996); ^*h*^Shih et al. (1997).*

**FIGURE 2:**
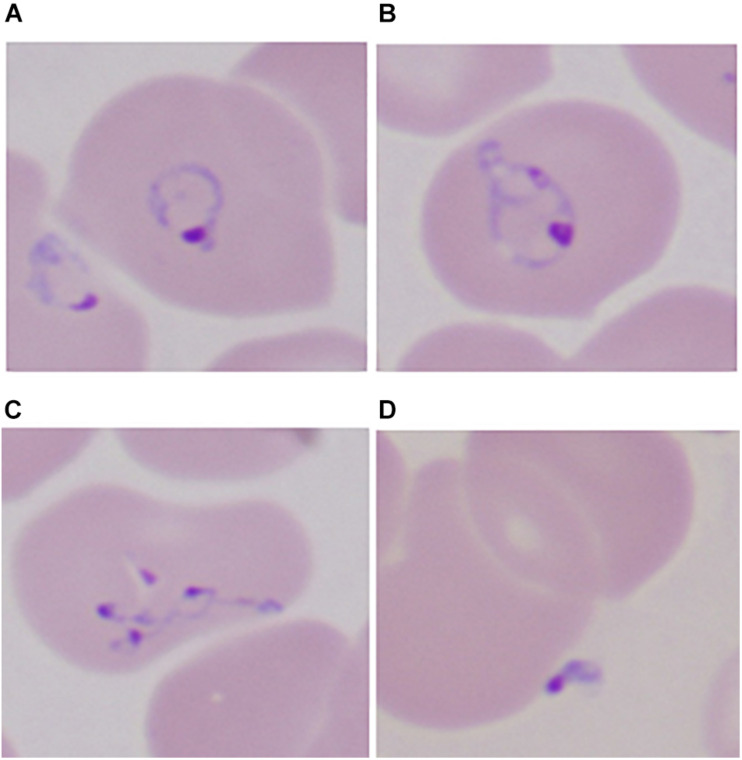
Developmental stages of intraerythrocytic *Babesia microti* parasite by Giemsa-stained thin blood microscopy. **(A)** Ring form with cytoplasm stained blue; **(B)** mature trophozoite with two small nuclei; **(C)** a tetrad (Maltese cross); and **(D)** an extracellular form. 1000× magnification. Adapted from Vannier and Krause (2012) (From The New England Journal of Medicine, Edouard Vannier, PhD., and Peter J. Krause, M. D., Human Babesiosis, 366, 2399. Copyright (2012) Massachusetts Medical Society. Reprinted with permission).

Most *Babesia* spp. are small (1–5 μm in length) and are pear-shaped, oval, or round with blue cytoplasm and red chromatin dots (DNA) when viewed on Giemsa-stained blood smears (Healy and Ruebush, 1980). Ring forms are the most common configurations noted on blood smears and are similar to those of *Plasmodium falciparum*. *Babesia* differ from *P. falciparum* by the absence of hemozoin pigment deposit in the ring form, the lack of schizonts and banana-shaped gametocytes, and the presence of tetrads (Maltese cross) (Figure 2) (Vannier and Krause, 2012). The latter are seldom noted but, if present, are pathognomonic of *Babesia*.

Babesiosis usually causes mild to moderate illness but asymptomatic infection may occur, and severe life-threatening disease occurs in immunocompromised hosts, such as those with asplenia, cancer, HIV/AIDS, the extremes of age (neonates and those over 50 years), and people taking immunosuppressive drugs (Falagas and Klempner, 1996; Hatcher et al., 2001; Froberg et al., 2004; Krause et al., 2008; Vannier and Krause, 2012; Menis et al., 2015; Dumic et al., 2020). Complications include acute respiratory distress syndrome (ARDS), severe anemia, congestive heart failure, disseminated intravascular coagulopathy (DIC), liver and renal failure, and shock (Hatcher et al., 2001; Krause et al., 2007; Dumic et al., 2020). We have a limited understanding of the pathogenesis of babesiosis but excessive proinflammatory cytokine production, cytoadherence of infected red blood cells, and severe anemia are posited as important pathologic mechanisms (Clark and Jacobson, 1998; Hemmer et al., 2000; Allred and Al-Khedery, 2004; Krause et al., 2007). Diagnosis is suspected on epidemiologic and clinical grounds and confirmed by blood smear and/or polymerase chain reaction (PCR; Krause et al., 2021). Treatment is generally effective with the use of atovaquone and azithromycin or clindamycin and quinine but may be problematic in highly immunocompromised hosts where acute fulminant disease may be fatal or prolonged antibiotic therapy may lead to antibiotic resistance (Krause et al., 2000, 2021; Wormser et al., 2010; Lemieux et al., 2016; Simon et al., 2017).

**FIGURE 3:**
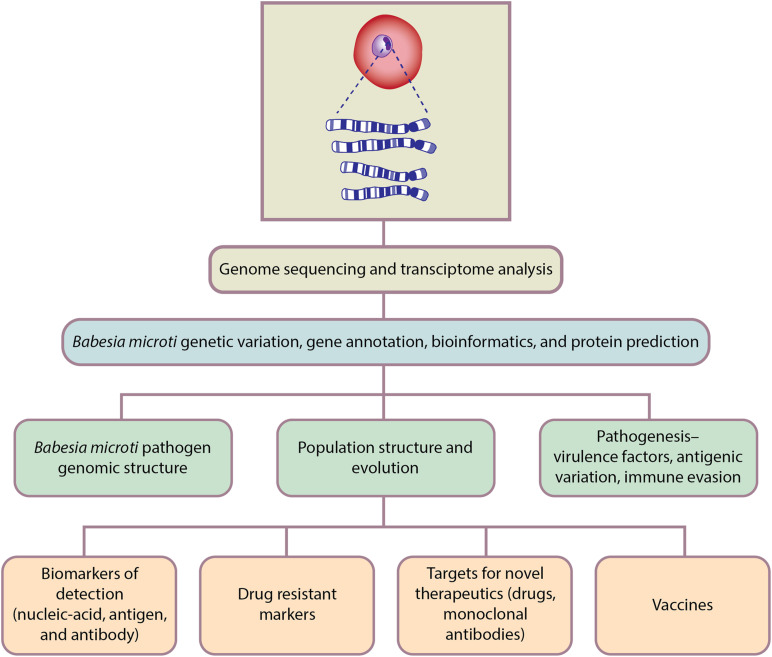
Genomics-based approaches for *Babesia microti* detection, molecular surveillance, understanding pathogenesis and developing therapeutics.

Genomics is the branch of molecular biology that focuses on the structure, function, evolution, and mapping of genomes. It aims at the characterization and quantification of all the genes and their interactions that affect the function of the organism. *B. microti* genomic studies have only recently been initiated but already have delivered important new insights regarding pathogen genomic structure, population structure, evolution, and pathogenesis; as well as biomarkers of detection, drug resistance markers, targets for novel therapeutics, and vaccines (Figure 3).

## *Babesia microti* Pathogen Genomics

### *Babesia microti* Genome Organization

The *B. microti* genome sequence was first published in 2012 (Cornillot et al., 2012). The *B. microti* genome is comprised of 4 chromosomes that contains approximately 6.5 megabase pairs (Mbp) that encode around 3,500 polypeptides, which is the smallest of all Apicomplexan genomes (Cornillot et al., 2012). A reannotation analysis of the *B. microti* genome sequence has identified 3,615 genes which encode for 3,567 proteins (Silva et al., 2016). A salient feature of the *B. microti* genome is the preponderance of unusually small introns that range in size between 18 and 21 base pairs, some of which are reported to be in frame, which is rare in eukaryotes (Silva et al., 2016). In contrast to the concentrated form of the mitochondrial genome in *Plasmodium* parasites, *B. microti* possesses a linear monomeric mitochondrial genome which contains a pair of unique repeats and a flip-flop inversion system. The flip-flop inversions may be involved in the switch on or off expression of the *B. microti* mitochondrial genes and gene fragments in their life cycle (Hikosaka et al., 2012).

In apicomplexan parasites, colocalization of virulence genes has been shown to influence spatial genome organization and is linked to virulence and survival (Bunnik et al., 2019). In *P. falciparum*, members belonging to multi-gene families that are responsible for virulence, antigenic variation, and immune evasion are colocalized in the genome (Reid, 2015; Bunnik et al., 2018, 2019). Studies of the comparative 3D genome organization of apicomplexan parasites has revealed that *B. microti* has a classical Rabl organization, which is similar to yeast and lacks a clear localization of virulent factor genes as seen with the *P. falciparum* parasite (Lenz and Le Roch, 2019). Thus, the *B. microti* genome organization is not strongly linked to the regulation of virulence genes.

### *Babesia microti* Phylogeny and Taxonomy

The apicomplexan clade of Hematozoa, which includes parasites infecting vertebrate hemocytes, is further divided into two major clades – the well-studied Haemosporidia, such as *Plasmodium*, and the Piroplasmida. Piroplasmida includes the traditionally defined genera such as *Babesia* and *Theileria*. Phylogenetic analyses based on the nucleus-encoded genes revealed that the recently described parasites/symbionts of the ascidian tunicates, *Cardiosporidium* (infecting *Ciona*) and *Nephromyces* (infecting *Molgula*) have emerged as a sister-group of all Hematozoa (Munoz-Gomez et al., 2019). These parasites are monoxenous (i.e., complete their life cycle in a single host species), unlike the crown group Hematozoa which have a definitive invertebrate host and a secondary vertebrate host (Munoz-Gomez et al., 2019). This suggests that the transition from monoxenous blood-parasitism in ascidians, a sister group of the vertebrates, to the dixenous parasitism typical of Hematozoa happened early in their evolution with the emergence of invertebrate blood-feeders that could serve as vectors. While piroplasms retain many of the features of this ancestral vertebrate blood parasite, species classified in the genus *Babesia* exhibit specialized modes of transmission, such as transovarial transmission from females to their offspring, as well as transstadial transmission between different stages in the life cycle of the tick vector. These features appear to have emerged as adaptations to their arachnid vectors.

Traditionally, piroplasms have been classified based on morphological/life-history features such as presence (*Theileria* and *Cytauxzoon*) or absence (*Babesia*) of schizogony; as well as size- “small” species (e.g., *B. microti* and *B. duncani*) versus “large” species (e.g., *Babesia bigemina*). However, a series of molecular phylogenetic studies over the past decade (reviewed in Jalovecka et al., 2019) using nuclear and apicoplast sequences have shown that the traditional morphological taxonomy of *Babesia* and allied genera does not reflect their true evolutionary relationships. Notably, these studies have shown that the genera of *Babesia* and *Theileria* are not monophyletic and that the genus *Cytauxzoon* is nested with the lineages that have been assigned to the former two genera (Lack et al., 2012). Instead, these studies suggest that the *Babesia*-*Theileria*-*Cytauxzoon* complex can be divided into 10 monophyletic clades that encompass 14 reference species included in the genus *Babesia*, 9 species included in *Theileria* and 2 species included in *Cytauxzoon*. Piroplasms infect a wide range of eutherian mammals, marsupials (e.g., *Theileria* spp. Clade IV), monotremes (*Theileria ornythorhynchi*, Clade II) and birds (e.g., *Babesia ugwidiensis*, Clade V) (Schnittger et al., 2012). While reptilian-parasitic piroplasms, such as Sauroplasma and Serpentoplasma have been reported (Adl et al., 2019), their relationship to the above complex remains unclear. Nevertheless, the widespread presence of the *Babesia*-*Theileria*-*Cytauxzoon* complex in warm-blooded vertebrates suggests that they have likely emerged early in the history of these vertebrates and have repeatedly jumped hosts (Jalovecka et al., 2019).

Consistent with this possibility, human parasitism among piroplasms has emerged on multiple occasions, most likely through accidental extension of the parasite host range due to interactions with ticks feeding on infected domesticates or wildlife. Of these, the *B. microti*-like group, defines “clade I,” which is the earliest branching clade of the *Babesia*-*Theileria*-*Cytauxzoon* complex. This clade includes four distinct lineages within it: Clade Ia parasites that infect rodents, monkeys and humans (e.g., *B. microti*; the primary agent of human babesiosis); Clade Ib parasites that infect canids and mustelids (e.g., *Babesia vulpes*); Clade Ic parasites that infect rodents (e.g., *Babesia rodhaini*); and Clade Id parasites that infect cats (e.g., *Babesia felis*). Like other piroplasms, clade I *Babesia* show transstadial transmission but lack transovarial transmission typical of the “classic *Babesia*” of clade X (*Babesia* species “sensu stricto”). In addition to the Clade I *B. microti*-like group, clades of other human-infecting piroplasms include members of Clade III (the “Western group”) with representatives like *B. duncani*, Clade IX comprised of *Theileria* sensu stricto (e.g., *T. annulata*, *T. parva*, and *T. orientalis*), and Clade X consisting of *Babesia* sensu stricto (e.g., *B. divergens* and *B. venatorum*). With over 90 species infecting a variety of wild and domestic mammalian hosts (Schnittger et al., 2012), several piroplasms have a significant worldwide veterinary economic impact. These include: (1) the bovine-infecting species, e.g., *B. bigemina*, *B. bovis*, and *B. divergens*; (2) the equid-infecting species, e.g., *Theileria equii* and *Babesia caballi*; (3) sheep/goat-parasites, e.g., *Babesia ovis* and *B. crassa*; and (4) carnivoran parasites, e.g., *B. felis* and *B. canis* (for review see Schnittger et al., 2012).

Given the distinct features of the *B. microti* and *B. microti-*like group from the *Babesia* sensu stricto group, it has been argued that in a future taxonomic revision the *B. microti* and *B. microti*-like group should be elevated to a separate genus along with the other distinct clades identified in recent studies (Rudzinska, 1976; Rudzinska et al., 1976; Goethert and Telford, 2003; Cornillot et al., 2012; Lack et al., 2012; Schnittger et al., 2012; Jalovecka et al., 2019). Additional studies are needed that include the creation of a large database of whole genome sequencing of *Babesia* spp. This will provide a complete examination of the genetic diversity of *Babesia* spp. that have such a significant worldwide public health impact.

## Genetic Variability in *Babesia microti* Populations

Genomic studies over the past decade have refined our understanding of the epidemiological characteristics of *B. microti*. In one study, genome sequence analyses of 42 *B. microti* samples from around the world show extensive genetic diversity (Lemieux et al., 2016). *B. microti* isolates from within the continental United States show a relatively stable genetic structure but these isolates possess significant genetic diversity compared with samples from geographically distant Alaska, Russia, and Japan. In the continental United States, two distinct genetic *B. microti* sub-populations were noted in the Northeast and Midwest regions. The authors predicted that these two parasite lineages entered the continent at different time points separated by more than 700 years (Lemieux et al., 2016). In the same study, parasite variants containing amino acid substitutions in the rp14, a subunit of riboendonuclease, were associated with relapsing disease. Additionally, genetic mutations in the atovaquone-binding regions of cytochrome b and the azithromycin-binding region of ribosomal protein subunit L4 were also identified. Based on the available whole genome wide sequence data, a 25 single nucleotide polymorphism (SNP) barcode was subsequently developed that supported the previous findings and identified two distinct *B. microti* lineages in the northeastern and midwestern United States (Baniecki et al., 2019).

The genetic diversity and population structure of *B. microti* parasites in the endemic regions of the northeastern United States has been characterized by Carpi et al. (2016). They employed the multiplex capture platform for characterizing genome-wide diversity and genetic relatedness in 25 *B. microti* isolates obtained from tick-vectors and humans. Their results showed that in the northeastern United States, *B. microti* was strongly structured into three highly differentiated genetic clusters. Interestingly, analyses of the apicoplast genome indicated that in the Northeast, the current genetic diversity in *B microti* dates back 46,000 years with evidence of population expansion in the past 1,000 years (Carpi et al., 2016). In another study, a total of 228 *B. microti* patient isolates from the New England area were genotyped by using variable number tandem repeat markers. Their results also showed the presence of three distinct *B. microti* population structures with each dominated by a single ancestral type (Goethert et al., 2018). The authors concluded that *B. microti* parasites prevalent in the northeastern United States have expanded from a common ancestral origin(s) on the mainland and not from Nantucket or other islands off the New England mainland where babesiosis was first reported to be endemic.

In summary, the genetic variability of *B. microti* in the United States is limited and different from *B. microti* in other parts of the world. *B. microti* can be divided into northeastern and midwestern groups with further division of three genetic clusters in the Northeast. It remains to be determined whether these closely related but different genetic groups are associated with different clinical severity.

## *Babesia microti* Antigenic Polymorphism and Immunodominant Antigens

The publicly available whole *B. microti* genome sequence database is still limited and the majority of information comes from parasite isolates collected in endemic areas in United States. In one study, genomics and gene expression profiling experiments were performed to identify polymorphic genes involved in host–parasite interactions. Analyses based on seven *B. microti* isolates from the northeastern United States revealed that antigenic polymorphism is generally restricted to a small number of highly expressed genes which belonged to the parasite surface proteome and secretome (Silva et al., 2016). Among the 3568 protein coding genes, 205 genes carried a total of 257 amino acid altering mutations, 27 of which contained nearly all mutations (Silva et al., 2016). It is possible that these surface expressed antigens are major immune targets and subjected to antigen variation that allows the parasite to escape host immunological surveillance and establish long term chronic infection.

In the past two decades, several groups have made efforts to identify and characterize immunodominant *B. microti* antigens to study their potential role as mediators of immunity and pathogenesis and for diagnostic and vaccine use. The majority of currently available antigens have been identified by screening *B. microti* genomic expression libraries against sera from infected mice or humans (Lodes et al., 2000; Homer et al., 2003; Xu et al., 2018; Zhou et al., 2018; Verma et al., 2020). The most prominent among these proteins are the diverse polymorphic multigene antigens termed the BMN (*B. microti* MN1 strain seroreactive antigen) family of antigens. Sequence analyses revealed patient-to-patient variation in the number and location of the sequence repeats within the BMN1-6 homolog. Furthermore, parasite isolates from nearby geographical locations were genetically similar compared to those from geographically distant locations (Lodes et al., 2000).

In two recent reports, immuno-screening of protein arrays based on *B. microti* antigen expressed in *Escherchia coli* has led to identification of several immuno-reactive antigens, some of which were previously uncharacterized (Xu et al., 2018; Zhou et al., 2018). In another study, Elton et al. (2019) employed a mammalian expression system to identify 54 genes that are predicted to encode surface and secreted *B. microti* blood stage antigens. The recombinant antigens produced were conformationally dependent and were used to measure the kinetics of antibody responses during early and late phase *B. microti* infection in BALB/c mice. Antibody responses against several antigens were noted during the entire course of infection. While some mice were able to mount antibody responses against all immunoreactive antigens, other mice generated antibody responses against only a subset of antigens. These results are surprising because the mice used in the study were major histocompatibility (MHC) compatible. Some immunoreactive antigens continued to elicit antibody responses beyond day 125 following initial infection. Interestingly, a broadly similar profile of antibody reactivity was observed against the same antigen panel in serum samples obtained from mice that were infected with a genetically distant strain of *B. microti*. A heterogenic antibody response against a subset of these antigens also was observed in sera from babesiosis patients, although the sample size was too small to draw any firm conclusions.

Several studies have applied computational and bioinformatics analyses to predict *B. microti* proteins that could be involved in host–parasite interactions, immune evasion, and potential targets of antigenic polymorphism. Using this approach, Silva et al. predicted 420 proteins that putatively belonged to the *B. microti* secretome (Silva et al., 2016). Next, a protein array was constructed consisting of 174 proteins that are predicted to trigger immune responses in hosts during infection. Immuno-screening using sera from *B. microti*-infected mice identified 30 highly antigenic proteins. Fourteen of 30 proteins, including the two most antigenic proteins, were a part of the secretome (Silva et al., 2016). In a subsequent study using a 17-plex protein array based on glycosylphosphatidylinositol (GPI)-anchored proteins, BmGP12 was identified as a potential biomarker for detection of past or current *B. microti* infections in laboratory and field captured mice, as well as in babesiosis patients and healthy residents living in New England (Cornillot et al., 2016).

In a more recent study of *B. microti* immunodominant antigens, genome-wide immune-screening led to the discovery of 56 novel *B. microti* antigens, many previously uncharacterized (Verma et al., 2020). Five immunodominant *B. microti* proteins that were identified in the study were cell-surface proteins possessing antigenic extracellular domains characteristic of adherence proteins that interact with host proteins. For instance, one of these is a previously uncharacterized protein with four copies of the epidermal growth factor (EGF) domains BmEGF1 (BmR1_03g00690). While EGF domain repeat proteins are widely known in Apicomplexa (Anantharaman et al., 2007), this protein is not closely related to any of them and its EGF domains share specific features in the pattern of conserved cysteines with certain proteins from *Giardia intestinalis* and animal secreted proteins (Verma et al., 2020). Consistent with this data, confocal microscopy studies showed that two of these antigens were expressed on the surface of infected erythrocytes, raising the possibility of their role as cytoadherence antigens. A combination of these two novel antigens and one previously described antigen provided 96% sensitivity and 100% specificity in detecting *B. microti* specific antibodies in babesiosis patients using an enzyme-linked immunosorbent assay (ELISA) platform (Verma et al., 2020).

Two of these five immunodominant proteins that are the most reactive *B. microti* antigens (BmR1_03g04855 and BmR1_03g00785) belong to the BMN class of proteins (Lodes et al., 2000; Homer et al., 2003; Silva et al., 2016; Xu et al., 2018; Zhou et al., 2018; Verma et al., 2020). A comprehensive analysis of these proteins shows that BMN class proteins do not constitute a monophyletic group and should not be considered as a unified “BMN family.” Instead, the majority can be classified into multiple evolutionarily unrelated groups of BMN proteins.

The first major group of BMN proteins generally correlate with the following families: (i) BMN1–10, N1–10, BMN1–4, BMN1–3B, BMN1–8, and BMN1–11 proteins from the *B. microti* MN1 strain, (ii) the IRA protein from the *B. microti* Gray strain and, (iii) the Br-1 and Br-2 proteins from the *B. rodhaini* Japanese strain. These proteins correspond in large part to the antigens termed BMN1 by Silva et al. (2016). *B. microti* R1 was found to possess 10 members of this group, including BmR1_03g04855 (Table 3 in Verma et al., 2020). They are characterized by one to five copies per protein of a domain with a N-terminal 8 β-strand sandwich, followed by a C-terminal disulfide bond-supported structure. Divergent versions of this domain are found outside of the *B. microti*-like strain, in a group of secreted proteins expanded across *Theileria*. Hence, this domain was named piroplasm β-strand (PiβS) domain.

The second group of BMN proteins is comprised of BMN1–2, BMN1–3, BMN1–6, BMN1–7, BMN1–9, BMN1–13, BMN1–4, MN-10, N1–21, all from the *B. microti* MN1 strain; BmSA1 from the *B. microti* Gray strain; BmP32 from the *B. microti* Munich strain; MSA1 and MSA2 from *B. rodhaini* Australia strain; and Br-1, p25, and p26 from the *B. rodhaini* Japanese strain. The Br-1, p25, and p26 proteins correspond in large part to the antigens termed BMN2 by Silva et al., 2016. Nine proteins in the second group of BMN proteins are present in the *B. microti* R1 strain (Table 3 in Verma et al., 2020). These are distinguished by a novel *B. microti*-like group-specific extracellular domain with 9 α-helices and a C-terminal hydrophobic GPI-anchor. Accordingly, it was named the *Babesia* α-helical cell surface (BAHCS) domain. In contrast to the PiβS domain, the BAHCS domain is always found in a single copy in a protein; however, on occasions both these domains might occur in the same protein (e.g., Br-1 from *B. rodhaini* and its cognate BMN1–4 from the *B. microti* MN1 strain). Both the PiβS and BAHCS domains shows a similar evolutionary tendency for independent lineage-specific expansions in *B. rodhaini* and *B. microti* along with notable inter-strain radiations in the later species (Verma et al., 2020). Beyond these, smaller BMN groups are formed by the paralogous BMN1–17 and BMN1–20 proteins and the Maltese cross form-related antigen (BMN1–15/N1–15) and BmR1_02g04285 (BmMCFRP1) that are unrelated to these groups (Verma et al., 2020).

The biological relevance of the antigenically polymorphic BMN family antigens and other immunodominant family antigens, as well as other adherence antigens in *B. microti* parasites, remains to be determined. Their rapid evolution in phylogenetically close lineages and independent lineage-specific expansions between them are the hallmark of proteins in an arms race with the host. This is reminiscent of other apicomplexan surface proteins such as the rifin-like and the var/DBL1 superfamilies in *P. falciparum*, and the vir/yir superfamilies in *Plasmodium vivax*/*P. yoelii* (Anantharaman et al., 2007). Future studies are needed to determine whether any of these antigens expressed on the surface of *B. microti*-infected red blood cells contribute to an immune escape mechanism that leads to persistent infections in animals and humans.

While data on *B. microti* proteins that are derived from genomic studies are now expanding, our knowledge of the parasite proteome based on direct protein profiling is very limited. By applying a combination of nanotechnology and mass spectroscopy (MS), Magni et al. (2019) have generated a proteome profile of intraerythrocytic *B. microti* parasites during acute phase infection in hamsters. They have identified ∼500 proteins with assigned functions, such as transport, carbohydrate and energy metabolism, signaling transport, mobility and invasion, and immune response. This proteome database could be exploited for novel diagnostic and vaccine targets and better understanding of parasite biology, host immunity, and pathogenesis. In a subsequent study, the nanotechnology-MS methods were applied to identify *B. microti* proteins in blood, serum and urine during infection in hamsters (Magni et al., 2020). A distinct but less abundant protein profile was observed in urine compared to blood and plasma. These studies highlight the potential value of the nanoparticle-MS methods to detect *B. microti* proteins in complex analytes such as blood and plasma.

Overall, data from whole genome, parasite genotyping, and expression profiling studies are helping to develop genetic tools for surveillance of acute and asymptomatic *B. microti* infections and for monitoring transmission in new endemic areas. Such studies are also expanding our knowledge of *B. microti* antigens that perform important functions, such as inducing protective immune responses and mediating host pathogenesis.

## *Babesia microti* Pathogenesis Genomics

The pathogenesis of *Babesia* spp. infections in humans is multifactorial, complex, and incompletely understood. In particular, molecular processes that underlie pathogenic mechanisms in human babesiosis have not been well researched. Pathogenesis studies are complicated by variation in pathogenic mechanisms for different *Babesia* spp. and variation in the immunologic status and pathophysiological response of hosts. Three clinical patterns of human babesiosis have been described: acute symptomatic disease which may be mild, moderate or severe, acute asymptomatic infection, and persistent infection (Vannier et al., 2008; Vannier and Krause, 2012). Acute asymptomatic infection is the most common clinical presentation. Asymptomatic infection occurs in about 20% of adults and is characterized by low parasitemia. Persistent infection follows acute symptomatic or asymptomatic infection and may last as long as 2 years (Krause et al., 1998, 2007; Raffalli and Wormser, 2016).

### Acute Disease

Several mechanisms may account for the severity of acute *B. microti* infection: an aberrant immune response that include excessive pro-inflammatory cytokine release, erythrocyte cytoadherence, persistent infection, and hemolytic anemia.

#### Aberrant Immune Response, Cytokine Storm

It is well recognized that immune responses that help protect the host against invading pathogens can also contribute to severe disease (Yokota, 2003; Huang et al., 2005). Over production of several pro-inflammatory cytokines during an array of infections is often associated with acute inflammation and tissue damage in the microenvironment of the lungs and other organs (Rubenfeld et al., 2005). The phenomenon of hyperimmune immune responsiveness leading to excessive cytokine production has been termed “cytokine storm” and is attributed to the most severe pathogenic consequences of infections such as SARS-CoV-2 infection (Fajgenbaum and June, 2020). Key cytokines involved in cytokine storm are TNF-α, IFN-γ, IL-1, IL-6, and IL-18. The major immune cell types involved are neutrophils, macrophages and NK cells.

Cytokine storm has been implicated in severe disease complications in babesiosis (Clark and Jacobson, 1998; Hemmer et al., 2000; Krause et al., 2007). Excessive TNF-α and IL-1 production by macrophages, Th1 lymphocytes, and other cells in response to high parasitemia, decreases capillary integrity and can cause multi-organ dysfunction, including ARDS (Hemmer et al., 2000). When the effect is more widespread with loss of intravascular fluid, hypotension and shock may result. Increased nitric oxide production following TNF-α and IL-1 release can help eradicate microbial pathogens but also cause tissue damage (Aguilar-Delfin et al., 2001; Vannier et al., 2015; Djokic et al., 2018). Elevated blood concentrations of TNF-α have been associated with the expression of adherence molecules ICAM-1 and VCAM-1 in vascular epithelium and with cerebral malaria in children (World Health Organization (WHO)., 2000; Krause et al., 2007).

The genomic underpinnings of cytokine storm have just begun to be elucidated. The dynamic of cytokine production leading to cytokine storm is complex and balanced by a number of factors, including proinflammatory cytokines and their cognate soluble receptor or inhibitors and the production of anti-inflammatory cytokines such as IL-10 (Tisoncik et al., 2012). Previous genomic studies have demonstrated the upregulation of proinflammatory cytokines in animal models during influenza infection (Kash et al., 2006; Kobasa et al., 2007; Cilloniz et al., 2009). The signature cytokine genes showing strong upregulation include IL-6, IL-8, CCL2, CCL-5, CXCL10, and CXCL9 (Baskin et al., 2009; Zhou et al., 2020). More recently, genomic databases generated from transcriptome analyses of cytokines, chemokines and immune cells during acute infections, such as those caused by influenza virus, dengue viruses, SARS-CoV-2, and other pathogens have begun to provide new insights regarding events leading to cytokine storm (Afroz et al., 2016; Hancock et al., 2018; Zhou et al., 2020).

Other mechanisms that influence cytokine-mediated regulation of severe disease include the association of TNF-α promoter polymorphisms (G-238A and G-308A) with susceptibility to diseases as diverse as systemic lupus erythematous and *P. falciparum* malaria (Mahto et al., 2019), as well as epigenetic regulation of cytokine storm in COVID-19 patients (Sawalha et al., 2020). Carefully designed transcriptome and cytokine profiling studies in severe babesiosis patients and in chronic asymptomatic *Babesia* infections would help to identify the molecular factors that lead to severe disease with fatal consequences in susceptible individuals. Such studies may help to develop immunotherapies that could ameliorate the most severe *B. microti* complications, such as hemolytic anemia, ARDS and kidney damage in babesiosis patients.

#### Erythrocyte Cytoadherence

Residence within red blood cells offers some protection for *Babesia* against host immune factors but *Babesia* infected erythrocytes are recognized and removed in the spleen. Cytoadherence of *Babesia*-infected erythrocytes to vascular endothelium is thought to allow the pathogen to complete its life cycle, leave the erythrocyte briefly, and infect another red blood cell without ever traversing the spleen. Excessive *Babesia*-induced erythrocyte adherence may contribute to babesiosis complications. *Babesia*-induced red cell cytoadherence has been associated with *B. bovis* and *B. duncani* induced pathology (Clark et al., 2006a; Usmani-Brown et al., 2013; Allred, 2019). *B. bovis* strains with increased cytoadherence activity *in vitro* have increased virulence in cattle (O’Connor et al., 1999). When parasitemia is high in *B. bovis*-infected cattle, a large number of infected red blood cells may adhere to small capillaries in the brain, causing vascular obstruction, anoxia and death of neurons. This is associated with the same stroke syndrome that is seen with cerebral malaria. Cytoadherence also has been associated with lung injury in *B. duncani* infected hamsters and mice (Dao and Eberhard, 1996; Hemmer et al., 1999, 2000; Krause et al., 2007), as well as renal injury in *B. bovis*-infected cattle (Patarroyo et al., 1982; Everitt et al., 1986). It remains unclear whether *B. microti* induces red blood cell cytoadherence. Vascular stasis and blockage has been demonstrated in the retina of a human patient infected with *B. microti* but another single case study failed to demonstrate evidence of *B. microti*-infected red blood cell cytoadherence to vascular endothelium in the brain of a comatose patient (Ortiz and Eagle, 1982; Clark et al., 2006b; Ortiz et al., 2020).

The genomic etiology of *Babesia* infected red blood cell cytoadherence has been described for *B. bovis*. A multigene family, *ves1*α, in *B. bovis* is responsible for the production of adherence variant erythrocyte surface antigen 1 or VESA1 (Allred et al., 2000). This family of variant proteins are found on the surface of *B. bovis*-infected erythrocytes. A similar multigene family (*var* genes) is found in *P. falciparum* that encodes for the production of *P. falciparum* erythrocyte membrane protein 1 or PfEMP1 (Jensen et al., 2020). These surface proteins mediate infected erythrocyte adherence to vascular endothelium, which make *Babesia* and *Plasmodia* less accessible to attack by host immune cells (Allred and Al-Khedery, 2006; Krause et al., 2007). In a recent study, several adherence proteins have been found on the surface of *B. microti*-infected red blood cells in laboratory mice (Verma et al., 2020). These findings support the possibility that *B. microti*-induced cytoadherence may contribute to disease complications.

### Persistent Infection

Persistence of infection is critical for the survival of *Babesia* as they rely on transfer between rodent host and tick vector. Once infected, both the primary host (*Peromyscus leucopus*) and tick vector (*I. scapularis*) remain infected for life, increasing the chance of transfer of infection from one to the other. The same mechanism(s) that ensure persistence of infection in wildlife are likely to be operative in humans as they too experience persistent infection for months, even though they are dead end hosts (Spielman et al., 1981; Telford and Spielman, 1993; Krause et al., 1998; Moritz et al., 2016). At least three mechanisms are thought to contribute to persistence: intraerythrocytic location, cytoadherence, and regulation of parasite release from the erythrocyte.

The intraerythrocytic location of *Babesia* protect them against host immune factors. Cytoadherence of *Babesia*-infected erythrocytes to vascular endothelium avoids splenic destruction of *Babesia*. Variable parasite release from the erythrocyte also contributes to persistent *Babesia* infection. The work of Lobo and colleagues has shown that the intraerythrocytic life cycle of *B. divergens* is flexible and that egress from the erythrocyte can occur rather quickly after red blood cell invasion or later in infection. Once established within the red blood cell, early egress from erythrocytes would be favored when there is a strong need to expand the population, as occurs early in infection. Later egress following several cell divisions is more likely when infection is well established and the intravascular environment becomes hostile due to host immune activation and antimicrobial therapy (Lobo et al., 2019). It only takes a few infected erythrocytes to support persistence of infection in a host, which helps explain the recrudescence of infection that can occur in immunocompromised hosts despite low level parasitemia after prolonged (6 weeks or longer) antimicrobial therapy (Krause et al., 2008).

The clinical consequences of persistent infection include relapsing disease in immunocompromised individuals and transfer of *Babesia* to blood transfusion recipients following donation from an asymptomatic infected blood donor. A prospective follow-up study of 46 babesiosis patients demonstrated that *B. microti* parasitemia can persist for months with or without anti-*B. microti* therapy (Krause et al., 1998). Blood samples were obtained from patients during acute infection and every three to 6 months thereafter for amplification of *B. microti* DNA using PCR. About half (*n* = 22) of the patients were treated with clindamycin and quinine. *Babesia* DNA persisted asymptomatically in these patients for 2–13 months, depending in part on whether parasitemia duration was measured by the last recorded PCR positive sample or the first PCR negative sample. Asymptomatic infection persisted even longer in a group of patients who had mild *B. microti* infection and were not treated because of concern about side effects of clindamycin and quinine, the only effective therapy available at the time of the study. In these cases, parasitemia duration was 7–27 months. Only one of the 46 previously healthy patients had recrudescence of infection and that occurred 27 months after initial diagnosis (Krause et al., 1998). In contrast, recrudescence is more common in highly immunocompromised patients, especially those with defective antibody production (Krause et al., 2008). Prolonged *Babesia* disease has been described in immunocompromised hosts, with relapsing symptoms lasting up to 9 months and parasitemia continuing for more than 2 years (Krause et al., 2008; Raffalli and Wormser, 2016; Allred, 2019; Bloch et al., 2019). These patients are markedly immunocompromised with underlying diagnoses that include HIV/AIDS, malignancy, and asplenia. A retrospective case series of consecutively enrolled babesiosis patients who failed to respond to standard anti-*Babesia* antibiotic therapy also demonstrated that patients with these immunosuppressive conditions experienced persistent and relapsing babesiosis (Krause et al., 2008). Interestingly, 10 of the 14 patients in this series suffered from B cell lymphoma and had been treated with Rituximab, an anti-B cell monoclonal antibody. These data suggest that an impaired anti-*Babesia* antibody response, along with generalized immunosuppression, prevents clearance of *B. microti* infection. Long term antibiotic therapy of at least 6 weeks with at least 2 weeks of negative blood smears, rather than the standard 7–10 days, was required to resolve infection in these severely immunocompromised patients. Severe and persistent *B. microti* infection has been associated with advanced age in a mouse model and in humans (Vannier et al., 2004; Vannier and Krause, 2012).

Asymptomatic persistence of *Babesia* infection accounts for transmission of *Babesia* through blood donation. In the United States, *B. microti* is one of the most common pathogens transmitted by blood transfusion. Since 1979, there have been more than 250 cases of transfusion-transfusion transmitted babesiosis (Herwaldt et al., 2011; Moritz et al., 2016; Linden et al., 2018; Gray and Herwaldt, 2019). Furthermore, data from several studies, including the national babesiosis surveillance program that tracks clinical cases, indicate that both the clinical burden and foci of transmission of *Babesia* are expanding, which also means increased transfusion transmitted babesiosis risk to the U.S. blood supply (Menis et al., 2015; Linden et al., 2018; Gray and Herwaldt, 2019). Lack of knowledge of the mechanisms of persistence of parasitemia in asymptomatic carriers, and the intraerythrocytic characteristics of the parasite, present unique challenges in identifying *Babesia* infection in blood donors. While the minimum infectious dose to transmit *Babesia* in humans is not known, results from a mouse model suggest that as few as 10–100 infected RBC are sufficient to establish fulminant blood stage infection (Bakkour et al., 2018).

Limited epidemiological surveys in *Babesia*-endemic states using laboratory-based nucleic-acid-based tests (NAT) and antibody testing have provided important information about the percentage of asymptomatic infected donors, the relationship between seropositivity and parasitemia, and the seasonality of transmission in endemic areas (Leiby et al., 2005, 2014; Johnson et al., 2009). In more recent years, results from prospective studies in large cohorts of blood donors conducted under the Investigational New Drug protocols have shed light on the prevalence of *B. microti* infections in asymptomatic healthy adults in endemic areas and non-endemic states (Levin et al., 2016; Moritz et al., 2016; Tonnetti et al., 2020). Results showed one confirmed infected sample per 1331 donations in endemic states (67 positives/89,153 donations) (Moritz et al., 2014) and one confirmed positive per 2351 donations (211 positives/496,270 donations) (Tonnetti et al., 2020) in endemic states plus Florida using investigational NAT assays. Cumulative results from the surveillance programs (Herwaldt et al., 2011; Linden et al., 2018; Gray and Herwaldt, 2019) and donor testing studies (Johnson et al., 2009; Moritz et al., 2016) have revealed that while tick-borne infections are seasonal, transfusion transmitted cases occur year-round, although they too peak during the tick-transmission season. Transfusion transmitted babesiosis cases are also reported outside the bounds of recognized endemic states due to travel to endemic areas from non-endemic areas and import of blood products from endemic to non-endemic states (Cangelosi et al., 2008; Ngo and Civen, 2009).

### Hemolytic Anemia and Hypercoagulability

After invasion of erythrocytes, *B. microti* multiply by binary fission, resulting in two to four daughter cells (merozoites). Rupture of erythrocytes that occurs with merozoite release is associated with fever, anemia, jaundice, hemoglobinemia, hemoglobinuria, tissue hypoxia, and renal insufficiency (Vannier and Krause, 2012; Vannier et al., 2015). Malaria merozoite egress from erythrocytes is synchronous while *Babesia* merozoite egress is non-synchronous. Synchronous release leads to paroxysms of fever interspersed with periods of apparent wellness, whereas non-synchronous release results in a more continuous pattern of fever. Because non-infectious hemolytic processes do not cause fever, additional factors associated with the lysis of the red cell are thought to result in febrile episodes. Hemolytic anemia with hypoxia has been implicated in exacerbation of congestive heart failure. Red cell membrane debris may lead to sequestration, vascular stasis, and functional impairment in the kidney and possibly other organs. Renal impairment is commonly noted, including renal failure in about 5% of babesiosis patients (Persing et al., 1995; Hatcher et al., 2001). Hemolytic anemia due to production of autoantibodies 2–4 weeks after the diagnosis of babesiosis has been described in a subset of asplenic patients who had no previous history of autoimmunity (Woolley et al., 2017). The genetic aspects of the severity of babesiosis-induced hemolytic anemia and hypercoagulability have not been investigated.

## Conclusion

Genome sequencing of *Babesia* parasite isolates from diverse geographic locations has improved our understanding of genetic diversity of *Babesia* and provided genetic tools to monitor the areas of transmission and expansion where the disease had not previously existed. Genomic studies also have helped elucidate the pathogenesis of *B. bovis* and *B. divergens* infections that may have relevance for all pathogenic *Babesia* species. Such studies also have provided potential therapeutic and vaccine targets. Computational analyses and biological characterization of novel *B. microti* antigens has made possible the assignment of functions and elucidation of biological pathways that could provide new diagnostic tools and novel drug and vaccine targets. Availability of superior detection assays will allow improved diagnosis for acute babesiosis and help protect the blood supply against transfusion-transmitted babesiosis. Additional research is needed to improve our knowledge of parasite invasion, cell cycle and proliferation, and factors that mediate host immunity and pathogenesis. Finally, genomic studies should help in the development of novel treatment options. These include new drugs and/or biologics such as monoclonal antibodies that are urgently needed for treatment of those patients experiencing severe babesiosis in whom standard anti-*Babesia* antibiotics are not effective.

## Author Contributions

SK and PK conceptualized the review, analyzed the data, and helped to write the manuscript. AP, SB, LA, and SM helped to write the manuscript and prepared the figures. All authors read and approved the final manuscript.

## Conflict of Interest

The authors declare that the research was conducted in the absence of any commercial or financial relationships that could be construed as a potential conflict of interest.

## Publisher’s Note

All claims expressed in this article are solely those of the authors and do not necessarily represent those of their affiliated organizations, or those of the publisher, the editors and the reviewers. Any product that may be evaluated in this article, or claim that may be made by its manufacturer, is not guaranteed or endorsed by the publisher.
